# Widespread pain – do pain intensity and care-seeking influence sickness absence? – A population-based cohort study

**DOI:** 10.1186/s12891-016-1056-1

**Published:** 2016-05-04

**Authors:** Søren Mose, David Høyrup Christiansen, Jens Christian Jensen, Johan Hviid Andersen

**Affiliations:** Danish Ramazzini Center, Department of Occupational Medicine, Regional Hospital Herning, Gl. Landevej 61, DK-7400 Herning, Denmark; VIA Faculty of Health Sciences, Physiotherapist College, Gl. Struervej 1, DK-7500 Holstebro, Denmark

**Keywords:** Musculoskeletal pain, Widespread pain, Pain intensity, Care-seeking, Sickness absence, Cohort study

## Abstract

**Background:**

Both musculoskeletal pain-intensity in relation to a specific location (e.g. lower back or shoulder) and pain in multiple body regions have been shown to be associated with impaired function and sickness absence, but the impact of pain intensity on the association between widespread pain and sickness absence has not been studied. Additionally it is unknown whether care-seeking in general practice due to musculoskeletal disorders has a positive or negative impact on future absenteeism.

The purpose of this study was to examine the influence of pain intensity on the association between number of musculoskeletal pain sites and sickness absence, and to analyze the impact on absenteeism from care-seeking in general practice due to musculoskeletal disorders.

**Methods:**

3745 Danish adults registered with eight General Practitioners (GPs) in one primary medical center reported location and intensity of experienced musculoskeletal pain in seven different body regions in February 2008. Outcome was duration of sickness absence based on register data divided into long-term (>52 weeks during follow-up) and sickness absence of shorter duration (12–52 weeks during follow-up) over a period of 4 years. Data on pain-intensity were analyzed at three different cut-off levels for each body region: i) > 1 (any pain), ii) > 2 (bothersome pain), iii) > 3 (very bothersome pain). Analyses were stratified and compared between participants without GP contact and participants with GP contact due to musculoskeletal disorders.

**Results:**

Musculoskeletal pain in more than two body regions was strongly associated with long-term sickness absence in an exposure-response pattern. Different cut-off levels of pain intensity and adjustment for age, sex, educational level and work environmental factors did not alter the results. Similar findings were observed for sickness absence of shorter duration, although the association was weaker. Care-seeking in general practice due to musculoskeletal disorders did not overall alter the odds of later sickness absence.

**Conclusion:**

Pain intensity and care-seeking due to musculoskeletal disorders did not seem to influence the association between the number of pain sites and later sickness absence. The number of musculoskeletal pain sites seems to be a strong risk factor for later sickness absence.

## Background

Musculoskeletal disorders are one of the leading causes of long-term sickness absence and health-related early retirement pension [1–4]. High level of musculoskeletal pain intensity is strongly associated with impaired function and a prognostic factor for long-term sickness absence [5–10]. However, until recently, prognostic factors for poor outcomes have often been studied in relation to localized musculoskeletal pain or one clinical disorder (e.g. low back pain, shoulder pain), even though several recent studies have shown that localized pain is relatively rare, and musculoskeletal pain often occurs in various body regions simultaneously [3, 11, 12]. Furthermore, pain in multiple body regions (widespread pain) are substantially differently associated with other risk factors than localized pain. Gender, age, symptoms of stress and physical strain have demonstrated significantly stronger association with widespread pain compared to localized pain [13] and functional consequences increase proportionally with the number of body regions with musculoskeletal pain [12]. This indicates that studies analyzing musculoskeletal pain should take number of body regions with pain as well as pain intensity into account. However, the association between pain intensity and the number of body regions with pain has not been thoroughly studied, even though a recent study indicates that these factors increase proportionally [14].

Widespread pain has been associated with sickness absence in previous population studies [3, 15, 16]. Kamaleri et al. found a strong association between widespread pain and risk of self-reported long-term sick leave and early retirement pension 14 years later [3]. Similar results were found by Haukka et al. [15]. Participants with widespread pain at baseline had a significantly higher degree of persistent sickness absence than those with localized or no pain [15]. As no information about pain intensity was reported in these studies, the impact of pain intensity on the association between widespread pain and sickness absence remains unclear. One could expect that minor symptoms with a significant effect on daily life or quality of life might be included and consequently weakened the association. Alternatively, the number of body regions and pain intensity might increase proportionally and thereby partly measure the same underlying concept, thus the intensity of pain might not be as important as the fact that pain is present.

Similarly, the impact of care-seeking in general practice on the consequence of musculoskeletal pain has not yet been investigated. In Denmark long-term sickness absence has to be approved by the General Practitioner (GP), indicating that long-term sickness absence and care-seeking is associated. It could be expected that care-seeking might simply be an indication of the severity of the condition, and as a consequence care seeking has a significant higher risk of sickness absence. However, previous studies have shown that only a subgroup of people with musculoskeletal pain seek treatment [17, 18], and care-seeking is not a natural matter of course following musculoskeletal pain. Furthermore other symptoms than pain (e.g. somatization) only partly predicts care-seeking in general practice [19]. Whether sickness behaviour with self-management or sickness behaviour with care-seeking in general practice due to musculoskeletal disorders has the most positive impact on absenteeism has not been investigated.

The aims of the study were 1) to examine the impact of pain intensity on the relationship between number of body regions with musculoskeletal pain and sickness absence and 2) to investigate whether care-seeking in general practice due to musculoskeletal disorders has an impact on absenteeism.

## Methods

### Study population

The study is a register based follow up study on a previously established cohort in the municipality of Odder, East Jutland, Denmark (The Odder project) investigating risk factors for care-seeking in general practice for back pain and upper extremity pain [19–21]. The study population is a subset of a representative cohort of Danish adults registered with eight different GPs at the same primary medical center. In total 8517 individuals between 17 years and 65 years were invited to participate and received a mailed or web-based self-administered questionnaire in February 2008. The questionnaire covered a wide range of information on individual, psychosocial and work-related factors. All participants signed written informed consent forms and the study was approved by the Danish Data Protection Agency (Project number 7-2007-03). According to Danish law, this type of study does not require approval by committees on biomedical research ethics.

In total 5097 individuals (59.7 %) returned the baseline questionnaire. Differences between responders and non-responders have been published elsewhere. The proportion of women and the mean age were higher among responders, and there were no major differences in care-seeking among responders and non-responders [19]. Additional 1323 individuals (15.5 %) were excluded from this analyzes of whom 1154 (13.5 %) received permanent social security payment due to illness at baseline or retirement pension during the follow-up period. 39 (0.5 %) individuals died and 23 individuals (0.3 %) stayed abroad more than 6 months during the follow-up period. 107 (1.3 %) individuals were excluded due to missing exposure values. Thus 3745 individuals were eligible for the analysis.

### Outcome

Information on sickness absence was obtained from “The Danish register-based evaluation of marginalized groups of individuals based on registered social public transfer payments” (DREAM) administered by The Danish National Labor Market Authority. DREAM contains information on all Danish citizens who have received social benefits or any other transfer income since July 1991. This information is recorded on a weekly basis and provides valid data regarding labor-marked status [22]. The outcomes in this study were divided into “long-term sickness absence” and “Sickness absence of shorter duration” during the 4 year follow-up period. Sickness absence was defined as receiving sickness benefits, rehabilitation benefit or social assistance due to illness for 12-52 weeks (Sickness absence of shorter duration) or more than 52 weeks (Long-term sickness absence). Participants with permanent reduced work-capacity receiving early retirement pension during the follow-up period was analyzed together with long-term sickness absence. Participants with Sickness absence of shorter duration were analyzed separately.

As DREAM registration of sickness absence takes place on a weekly basis, few days of absenteeism may appear in DREAM as several weeks. Furthermore, few days of sickness absence are not registered equally for all Danish citizens. Registration of sickness absence of shorter duration than 3 weeks in DREAM depends on whether or not the person concerned has an employment-paid sickness insurance, receives any other transfer income or is sickness absent related to pregnancy [23]. In addition, the Danish working population has on average almost nine days of sickness absence each year [24]. Thus registered sickness absence of shorter duration than 12 weeks during the follow-up period was disregarded and classified as no sickness absence.

### Independent variables

The primary exposure variable was the number of self-reported body regions with musculoskeletal pain the last 4 weeks. Pain intensity and location was measured with parts of the pain module of The Standard Evaluation Questionnaire (SEQ), which has previously been validated [25]. Participants were instructed to mark experienced pain the last 4 weeks on a pain manikin and report intensity of experienced pain, if any, on a numeric ranking scale (NRS) from one (no pain) to seven (worst imaginable pain) for the following seven body regions: Head or face, left upper extremity, right upper extremity, chest or abdominal region, upper or lower back, left lower extremity and right lower extremity. Number of painful body regions during the last 4 weeks were then summed and recoded to the following four categories: No pain, pain in 1–2 regions, pain in 3–5 regions or pain in 6–7 regions. To explore the impact of pain-intensity data were analyzed at three different pain intensity cut-off levels at each body region: i) > 1 (any pain), ii) >2 (bothersome pain), iii) > 3 (very bothersome pain).

### Care-seeking in general practice

Care-seeking in general practice due to musculoskeletal problems was based on information from the International Classification of Primary Care system (ICPC-2) in which GP’s registered contact cause and diagnosis on all patients. GP contacts based on personal appearance due to musculoskeletal disorders 18 month following baseline were included in the present study. ICPC-2 is validated and reliable for recording musculoskeletal disorders [26]. Contact frequency was dichotomized: participants without GP contact and participants with at least one GP contact.

### Demographic variables

Sex and age was derived from the Danish Civil Personal Registration (CPR) number. The CPR-number is a unique personal identification number assigned to all persons with a permanent residence in Denmark. Using CPR-number, it is possible to link data from one or more registers or from other sources with register-based information at an individual level [27]. The CPR-number contains information on sex and age. Age was analyzed as a continuous variable.

### Educational level

Education beyond ordinary school was recorded at baseline. One of six levels of education could be chosen. These were recoded into three categories according to the level of vocational education and training: i) “No education beyond ordinary school” or “One or more short courses”. ii) “Skilled worker” or “Short further education”. iii) “Medium-level further education” or “Higher further education”.

### Physical work environment

The physical workload at baseline was evaluated using four items of Dutch Musculoskeletal Questionnaire [28]. Two different dimensions of physical work load was assessed and included in the study: Heavy lifting and monotonous repetitive work. Both dimensions were dichotomized with a cut-off point at 75 % percentile due to distributional skewness.

### Psychosocial work environment

Psychosocial work environment was evaluated by parts of the Glostrup Questionnaire [29]. Six questions were scored on a scale from one to six and two supplementary questions were scored on a scale from one to five. The questions referred to topics on job demand, help and support from colleagues and management, job satisfaction, decision authority and satisfaction with management. Each question was dichotomized á priori based on response options wording to indicate risk of poor psychosocial work environment according to questionnaire guidelines. The questions were analyzed as single items.

### Data analysis

Variables from baseline questionnaire and records displayed in form of frequencies and percentages, percentiles or mean with standard deviation were used to describe the study population and compare participants with non-participants.

The association between exposure (number of body regions with pain) and outcome (sickness absence) were analyzed using multivariable logistic regression analysis. All estimates are presented as odds ratios (OR) with 95 % confidence interval (95 % CI). Data were primary analyzed and presented at three different pain-intensity cut-off levels in the following three models: Crude analysis (Model 1). Analysis stratified on participants without GP contact and participants with at least one GP contact due to musculoskeletal disorders 18 month following baseline, respectively (Model 2). Adjusted analysis in which all á priori selected variables were included (Model 3). The association was tested for interaction between exposure and sex, age and GP contact. Additionally, the same association was analyzed in an alternative logistic regression analysis, in which the association between number of body-regions with self-reported musculoskeletal pain and sickness absence was adjusted for the highest overall reported pain intensity, across all body regions.

The consequence of missing values was controlled by reanalyzing model 1 and model 2 for both outcomes with the same participants whom were included in the model 3. OR’s with 95 % Cl from both analyses were then compared.

Significance level was set at 5 %. All analyses were performed using STATA 12.1 (StataCorp LP, College Station, TX, USA). Assumptions about linearity between log odds of the dependent variable and continuous and categorical co-variables were controlled visually by scatterplots.

### Missing data

Missing values on pain intensity and location on one or two out of the seven questions was coded as “not exposed” on the assumption that the respective location did not posed any discomfort for the participant in question, whom therefore considered the question as irrelevant. Missing values on pain intensity and location above two out of the seven questions were excluded from the analysis.

## Results

### Missing values

A total of 218 participants (5 %) had complete or partial missing values on pain location and intensity. Of these participants with missing values on one or two questions were coded as “no pain” (*n* = 111). Of the 111 participants with one or two missing values 88 % had reported pain elsewhere.

Participants with more than two missing exposure values (*n* = 107) were excluded from the analysis. The 107 individuals excluded due to missing values on pain intensity and location had significantly higher level of education (*p* <0.001), less physical work (*p* <0.002) and demanding work (*p* = 0.02), were less dissatisfied with management (*p* = 0.02) and had more frequent long-term sickness absence (*p* = 0.001) than the group of participants.

Baseline characteristics of the 3745 participants are shown in Table 1. A total of 202 persons (5.4 %) had long-term sickness absence during the follow-up period, while 278 persons (7.8 %) had sickness absence of shorter duration. Using a pain-intensity cut-off level >1 (any pain) 9.7 % of the participants did not report any pain in the 7 body regions at baseline while 13 % had widespread musculoskeletal pain (6–7 regions). Among those with long-term sickness absence 3 % did not have any pain at baseline while 28.7 % had widespread musculoskeletal pain. For sickness absence of shorter duration figures were 9 % and 22.6 %, respectively. Using a pain-intensity cut-off level > 2 (bothersome pain), 30 % of the participants reported having no bothersome pain in any body region at baseline while 4.5 % had widespread pain. For Very bothersome pain (pain-intensity cut-off level > 3) figures were 50.1 % and 1.8 %, respectively. About half of the participants with both long-term sickness absence and sickness absence of shorter duration (53 % and 54 %) had no contact with their GP 18 month following baseline. More women than men had short and long term sickness absence during the 4 years of follow up. The average age of all participants was 43 years.

**Table 1:** Baseline characteristics of 3745 occupational active participants stratified by duration of sickness absence covering the years 2008 to 2012

	Total	Long-term sickness absence^a^	Sickness absence of shorter duration^b^
All participants, *n* (%)	3745 (100)	202 (5.4)	278 (7.8)
Number of regions with any pain (pain-intensity cut-off level > 1)			
0, *n* (%)	362 (9.7)	6 (3.0)	25 (9.0)
1–2, *n* (%)	1311 (35.0)	32 (15.8)	70 (25.2)
3–5, *n* (%)	1584 (42.3)	106 (52.5)	120 (43.2)
6–7, *n* (%)	488 (13.0)	58 (28.7)	63 (22.6)
Number of bothersome pain regions (pain-intensity cut-off level > 2)			
0, *n* (%)	1124 (30.0)	21 (10.4)	53 (19.1)
1–2, *n* (%)	1419 (37.9)	46 (22.8)	100 (36)
3–5, *n* (%)	1033 (27.6)	103 (51.0)	107 (38.5)
6–7, *n* (%)	169 (4.5)	32 (15.8)	18 (6.4)
Number of very bothersome pain regions (pain-intensity cut-off level > 3)			
0, *n* (%)	1877 (50.1)	39 (19.3)	95 (34.2)
1–2, *n* (%)	1170 (31.2)	64 (31.7)	106 (38.1)
3–5, *n* (%)	630 (16.8)	85 (42.1)	68 (24.5)
6–7, *n* (%)	68 (1.8)	14 (6.9)	9 (3.2)
GP contact frequency			
No contact, *n* (%)	2613 (69.8)	107 (53.0)	150 (54.0)
At least one GP contact, *n* (%)	1132 (30.2)	95 (47.0)	128 (46.0)
Covariates			
Sex. Women, *n* (%)	2091 (55.8)	149 (73.8)	179 (64.4)
Age (Years), median (25;75 percentile)	43 (34;51)	44 (35;52)	44 (35;51)
Educational level^c^			
Low, *n* (%)	1317 (36.7)	48 (25.5)	78 (28.1)
Medium, *n* (%)	1657 (46.2)	100 (53.2)	145 (52.2)
High, *n* (%)	613 (17.1)	40 (21.3)	41 (14.7)
Physical work environment^d^			
Heavy lifting at work, *n* (%)	1024 (30.0)	32 (20.1)	51 (20.2)
Repetitive work, *n* (%)	1079 (32.1)	51 (31.7)	76 (30.3)
Psychosocial work environment^e^			
High job demands, *n* (%)	1050 (30.7)	56 (34.4)	98 (37.7)
High workload, *n* (%)	864 (25.3)	51 (31.1)	82 (31.7)
Low decision authority, *n* (%)	761 (22.6)	52 (32.9)	58 (22.7)
Low degree of stimulating tasks, *n* (%)	389 (11.6)	34 (21.5)	31 (12.2)
Low job satisfaction, *n* (%)	308 (9.2)	31 (20.1)	22 (8.6)
Low satisfaction with management, *n* (%)	896 (26.9)	59 (38.8)	74 (29.0)
Low support form management, *n* (%)	905 (27.2)	57 (35.4)	73 (28.6)
Low support from colleagues, *n* (%)	712 (21.3)	44 (27.9)	47 (18.3)

^a.^≥ 52 weeks of recorded sickness absence or permanent reduced work capacity during follow-up

^b.^Between 12 and 52 weeks of recorded sickness absence during follow-up

^c.^Missing: *n* = 158. ^d.^Missing: *n* = 399. ^e.^Missing: *n* = 547

### Musculoskeletal pain and sickness absence

Table 2 presents the results of the analysis of number of body regions with musculoskeletal pain and sickness absence. The number of body regions with musculoskeletal pain was strongly associated with long-term sickness absence an exposure-response pattern (test for trend: *p* < 0.001) with a more than 12 fold increased odds of long-term sickness absence for bothersome pain and very bothersome pain among those who experienced widespread pain. Self-reported musculoskeletal pain in more than two regions did significantly increase the crude odds of long-term sickness absence regardless of the cut-off level for pain intensity (Model 1). GP contact due to musculoskeletal disorders 18 month following baseline did not significantly change the odds of long-term sickness absence (Model 2), even though some of the OR changed quite considerably.

**Table 2:** Uni - and multivariate logistic regression analysis of the number of body regions with bothersome musculoskeletal pain and other covariates in 2008 and sickness absence during the following four years

Long-term sickness absence (> 52 weeks of recorded sickness absence or permanent reduced work capacity during follow-up.)						
Number of body regions with musculoskeletal pain at baseline	Any pain^a^		Bothersome pain^a^		Very bothersome pain^a^	
	(Pain-intensity cut-off level > 1 (Range 1–7))		(Pain-intensity cut-off level > 2 (Range 1–7))		(Pain-intensity cut-off level > 3 (Range 1–7))	
Model 1 OR (95 % CI)^c^ *N* = 3745						
No pain	Ref.		Ref.		Ref.	
1–2 regions	1.48 (0.62–3.58)		1.76 (1.04–2.97)		2.73 (1.82–4.09)	
3–5 regions	4.26 (1.85–9.76)		5.82 (3.61–9.38)		7.35 (4.97–10.87)	
6–7 regions	8.00 (3.41–18.77)		12.26 (6.88–21.88)		12.22 (6.27–23.83)	
Model 2 OR (95 % CI)^d^ *N* = 3745	No GP contact (*n* = 2613)	At least one GP contact (*n* = 1132)	No GP contact (*n* = 2613)	At least one GP contact (*n* = 1132)	No GP contact (*n* = 2613)	At least one GP contact (*n* = 1132)
No pain	Ref.	Ref.	Ref.	Ref.	Ref.	Ref.
1–2 regions	1.32 (0.50–3.52)	2.13 (0.27–16.88)	1.53 (0.82–2.84)	2.36 (0.86–6.48)	2.57 (1.52–4.34)	2.57 (1.34–4.92)
3–5 regions	3.39 (1.35–8.53)	6.63 (0.90–48.85)	4.31 (2.42–7.68)	8.17 (3.23–20.68)	6.57 (3.91–11.04)	6.64 (3.58–12.32)
6–7 regions	4.64 (1.73–12.43)	15.59 (2.10–116.05)	9.09 (4.35–19.03)	17.61 (6.21–49.93)	14.37 (6.46–31.96)	8.32 (2.45–28.32)
Model 3 OR (95 % CI)^f^ *n* = 3002^g^						
No pain	Ref.		Ref.		Ref.	
1–2 regions	1.88 (0.56–6.33)		1.50 (0.80–2.82)		1.81 (1.10–2.99)	
3–5 regions	3.86 (1.19–12.51)		3.74 (2.07–6.77)		4.50 (2.75–7.38)	
6–7 regions	6.84 (2.05–22.80)		8.42 (4.12–17.21)		9.42 (4.17–21.27)	
Sickness absence of shorter duration (≥ 12 weeks and ≤ 52 weeks of recorded sickness absence during follow-up)^e^						
The number of body regions with musculoskeletal pain at baseline	Any pain^a^		Bothersome pain^a^		Very bothersome pain^b^	
	(Pain-intensity cut-off level > 1 (Range 1–7))		(Pain-intensity cut-off level > 2 (Range 1–7))		(Pain-intensity cut-off level > 3 (Range 1–7))	
Model 1 OR (95 % CI)^c^ *n* = 3556						
No pain	Ref.		Ref.		Ref.	
1–2 regions	0.77 (0.48–1.23)		1.56 (1.11–2.20)		1.95 (1.46–2.60)	
3–5 regions	1.17 (0.75–1.83)		2.56 (1.82–3.61)		2.60 (1.88–3.61)	
6–7 regions	2.24 (1.38–3.65)		2.93 (1.66–5.17)		3.52 (1.68–7.40)	
Model 2 OR (95 % CI)^d^ *n* = 3556	No GP contact (*n* = 2512)	At least one GP contact (*n* = 1044)	No GP contact (*n* = 2512)	At least one GP contact (*n* = 1044)	No GP contact (*n* = 2512)	At least one GP contact (*n* = 1044)
No pain	Ref.	Ref.	Ref.	Ref.	Ref.	Ref.
1–2 regions	0.80 (0.43–1.46)	0.60 (0.28–1.30)	1.50 (0.97–2.31)	1.56 (0.88–2.75)	2.00 (1.38–2.90)	1.62 (1.02–2.57)
3–5 regions	1.35 (0.76–2.39)	0.68 (0.33–1.42)	2.17 (1.38–3.42)	2.48 (1.44–4.27)	1.99 (1.23–3.21)	2.57 (1.59–4.15)
6–7 regions	2.00 (1.04–3.84)	1.66 (0.77–3.57)	2.72 (1.26–5.88)	2.64 (1.12–6.23)	3.26 (1.23–8.61)	3.73 (1.15–12.17)
Model 3 OR (95 % CI)^f^ *n* = 2878^g^						
No pain	Ref.		Ref.		Ref.	
1–2 regions	0.76 (0.44–1.31)		1.44 (0.97–2.14)		1.81 (1.30–2.53)	
3–5 regions	0.98 (0.58–1.65)		2.14 (1.44–3.20)		2.04 (1.37–3.02)	
6–7 regions	1.82 (1.03–3.23)		2.34 (1.23–4.44)		3.08 (1.33–7.11)	

^a^Test for trend: *p* < 0.001, ^b^Test for trend: *p* < 0.01, ^c^Crude analyses, ^d^Stratified analysis, ^e^189 participants with ≥ 52 weeks of sickness absence during the follow-up period are not included in this analyzes, ^f^Adjusted for: GP contact. Sex, age, educational level, physical and psychosocial work environment. ^g^Number of participants are reduced due to missing values

After adjustment for GP contact, sex, age, educational level, physical and psychosocial work environment (Model 3) the association between the number of body regions with musculoskeletal pain and long-term sickness absence remained significant for pain in more than two body regions for all pain intensity cut-off levels with an exposure-response pattern, even though the strength of the association decreased to OR (95 % CI) 6.84 (2.05–22.80) for any pain, OR (95 % CI) 8.42 (4.12–17.21) for bothersome pain and OR (95 % CI) 9.42 (4.17–21.27) for very bothersome pain among the group widespread pain. The number of participants with musculoskeletal pain changed according to cut-off level of pain intensity (Table 1) (Test for symmetry: *p* < 0.001). However analyzing data at different cut-off levels of pain-intensity showed the same pattern. No interaction was found between exposure and sex, age or GP contact, respectively (data not shown). Visual control for linearity between the dependent variable and continuous and categorical independent variables by scatterplots was satisfactory (data not shown).

The results from the analysis between the number of body regions with musculoskeletal pain and sickness absence of shorter duration are displayed in Table 2. Generally the associations showed the same pattern as long-term sickness absence but OR decreased. GP contacts (model 2) did not change the associations. For Any pain significance was only found for crude analysis (Model 1) and participants with “No GP contact” (Model 2) and only for participants with widespread pain. For Bothersome pain and Very bothersome pain significant associations were found with an exposure-response pattern for all three models. Analyzing the association between number of body-regions with self-reported musculoskeletal pain and sickness absence adjusted for the highest overall reported pain intensity, across all body regions revealed a strong association with an exposure-response pattern, as was the case for the primary analysis (data not shown). Sensitivity analysis revealed that missing values had no influence on the results (data not shown).

## Discussion

This population-based study showed that the number of body regions with musculoskeletal pain were strongly associated with long-term sickness absence with an exposure-response pattern during a 4 year follow-up period. Changing the cut-off level of pain intensity had no decisive impact on the results. Contacts to the GP due to musculoskeletal problems during the first 18 months after baseline did not significantly alter the association. Adjustment for GP contact, sex, age, educational level, physical and psychosocial work environment did not change the associations between widespread pain and sickness absence. The association between the number of body regions with musculoskeletal pain and long-term sickness absence remained statistically significant for pain in more than two body regions for all pain intensity cut-off levels with an exposure-response pattern. In general, the association between number of body-regions with pain and sickness absence of shorter duration showed the same pattern as long-term sickness absence, but with less strong associations. Adjusting the same association for the highest overall reported pain intensity across all body regions did not alter the results.

### Methodological considerations

This study has several methodological strengths. The prospective design ensured that information about musculoskeletal pain and other co-variables were collected without knowledge of the outcome, thus differential misclassification is unlikely. Information about outcome and GP contact was based on registers, ensuring complete follow-up and equal data quality. The use of registers prevents differential misclassification, since outcome data were collected independently of information about exposure. However some limitations should be taken into account interpreting the results of the current study. About 40 % of the original cohort did not return the baseline questionnaire or deselected participation. The proportion of men and the mean age were lower among non-responders and non-responders did less often have GP contact with upper extremity pain, but not with back pain [19]. Other studies have shown that non-participants more often are less educated, single with greater degree of comorbidities than participants [30–32], but prevalence differences between participants and non-participates do not necessarily have a decisive impact on the association between variables, thus the results and the conclusion may not be deceive altered [31, 33].

Sickness absence was extracted from the DREAM register, which does not contain information on the cause of sickness absence. Thus, determining whether or not absenteeism was related to musculoskeletal disorders was impossible based on the present data. However, in this population sample of working adults we do not think that other major illnesses have had any major influence on the results.

Registers are often made for administrative purposes and the quality of data is occasionally not suitable for all research purposes [34]. Due to this, the quality of DREAM data on sickness absence of shorter duration than 12 weeks were too heterogeneous for any meaningful conclusion, and therefore disregarded in this study. Furthermore, the intent of this study was to investigate sickness absence of duration beyond what is to be expected due to normal periodic sickness.

A total of 107 individuals (2.8 %) were excluded from all analysis due to missing values on musculoskeletal pain. Although, this group was different than the participants on several variables it is, however, unlikely that exclusion of this relatively small group had any impact on the results.

Participants were asked to report pain-intensity on a NRS from one to seven in seven different body-regions within the last 4 weeks, as described previously. Furthermore, participants reported pain distribution within the last 4 weeks on a pain manikin. Comparison of reported musculoskeletal pain distribution at baseline for each participant between the SEQ pain questionnaire and the SEQ-pain manikin showed relatively consistent results. Spearman’s correlation between the two issues was 0.74 (*p* <0.001) (data not shown). 45 % of participants reported same degree of pain distribution in the two questions. 34 % reported pain distribution which only differed by one body region indicating that misclassification of pain distribution in the present study only occurred to a limited extent.

In the fully adjusted analysis (Model 3) the number of participants was reduced due to missing values. The consequence of this was controlled by sensitivity analysis. Model 1 and model 2 were reanalyzed for both long-term and short-term sickness absence with the same participants whom were included in the model 3. This analysis did not alter any of the results (data not shown).

### Pain intensity, pain location and sickness absence

The results from the present study are consistent with previous studies analyzing the association between the number of musculoskeletal pain and sickness absence. In a Norwegian population Kamaleri et al. found that the number of body regions (ten regions) with pain or discomfort was a strong prognostic marker for long-term sickness absence and permanent reduced work-capacity [3]. Similar results were found among working-aged Fins [15]. Comparing the results of present study and these two previous studies is not straight forward. Both exposure and outcome was defined differently on the basis of both self-reported and register data. In addition, follow-up time were14 years and 7 years, respectively - compared to 4 years in the present study. The studies were conducted in different time periods (1990–2004, 2001–2008 and 2008–2013) characterized by economic growth and worldwide recession, which might have influenced absenteeism differently in the respective cohorts. Still three Nordic population-based cohort studies showed unanimously that the number of body regions with pain were strongly associated with later sickness absence and disability.

To our knowledge this is the first time the impact of pain-intensity on the association between the number of body region with pain and sickness absence has been studied. This study revealed that the relative strength of the association did not decisively depend on pain-intensity. The results indicate that pain intensity and the number of body-regions with pain partly measures the same underlying concept. Predicting later sickness absence the level of pain-intensity do not seem to be as important as the fact that pain is present. In previous studies this same phenomenon has been reported in relation to other self-reported data related to health status and personality [35, 36].

Future research should emphasize on investigating whether more affective components of musculoskeletal pain have more impact on the predictive power of widespread pain in future sickness absence, by using alternative pain measurement scales.

According to Hill’s indicative criteria for causality, several factors support the association between number of painful body-regions and sickness absence as causal [37]. However, other studies have shown that both neurophysiological adaptations and individual cognitive processes contribute to the development of widespread pain [38, 39]. This indicates that the number of body regions with musculoskeletal pain expresses much more than physical pain. Widespread pain is associated with other symptoms such as chronic fatigue, insomnia, cognitive dysfunction, dizziness, headache, stiffness in muscles and joints, etc. [40–42] and widespread pain is often part of a complex cluster of symptoms with varying degrees of co-morbidity. Thus, people with widespread pain differ from those without pain or local pain in various ways. This may be a possible explanation why widespread pain is so strongly associated with poor prognosis in different populations, and could indicate that widespread pain itself is not the direct cause of sickness absence and incapacity.

### GP contact and sickness absence

A part of the purpose of the present study was to investigate the impact of GP contact due to musculoskeletal disorders on sickness absenteeism. Data on care-seeking in general practice due to musculoskeletal problems 18 month following baseline were included. As no information about exposure after baseline was available, we consider it reasonable only to include GP contact due to musculoskeletal disorders in this period. Any contact past this point could most likely be related to any other exposure. GP contact was stratified into two groups. The analysis revealed no statistically significant the difference between the groups even though the estimates changed quite considerably. This could however be explained by residual confounding due to dichotomizing. Stratification of GP contact, where participants with 3 or more contacts constituted a separate group and analyzing GP contact as a continuous variable did however not alter the results (data not shown). However, some of the analysis revealed higher OR’s for those who have contacted their GP with musculoskeletal pain, indicating that contacts to the GP did not prevent later long-term sickness absence. It was to be expected that GP contact would be an indicator of the severity of the disorder, thus it was somehow surprising that GP contact did not have any clear positive impact on sickness absence. A possible explanation of this finding could be that participants with widespread musculoskeletal pain and possible comorbidities sought medical care for a variety of other reasons than musculoskeletal pain.

The Municipality of Odder is inhabited by 21 500 people, in the town of Odder and its rural surrounding, and is quite typical for the Danish population as such. The study population consisted of both men and women with an age range between 17 and 65 years, including both town and countryside inhabitants. Respondents were employed in a wide range of occupations giving a broad selection of work-related exposures. The generalizability of this population to the wider Danish population is considered to be good.

## Conclusion

The number of body regions with musculoskeletal pain is associated with later sickness absence. Pain intensity has no further impact on this association and the number of musculoskeletal pain sites seems to be a more important risk factor of later sickness absence. Whether or not participants in the present study chose to seek care by the GP due to musculoskeletal disorders did not seem to change the future risk of sickness absence.

### Ethics approval and consent to participate

All participants signed written informed consent forms as stated in the Methods section. This study was approved by the Danish Data Protection Agency (Project number 7-2007-03). According to Danish law, this type of study does not require approval by committees on biomedical research ethics.

### Consent for publication

Not applicable.

### Availability of data and materials

Data from Danish National Registers are available from the Danish National Health and Medicines Authority for researchers who meet the criteria for access to confidential data. Study datasets cannot be accessed by other researchers according to Danish regulations. Researchers who are interested to use the same data need to apply for data access directly at the Danish National Health and Medicines Authority (http://www.ssi.dk/English.aspx) and The Danish Data Protection Agency (https://www.datatilsynet.dk/english/the-danish-data-protection-agency/introduction-to-the-danish-data-protection-agency/). Interested researchers may contact the corresponding author of this article for further guidance on this procedure.

## Abbreviations

CI: confidence interval
CPR-number: Danish Civil Personal Registration number
DREAM: The Danish register-based evaluation of marginalized groups of individuals based on registered social public transfer payments
GP: general practitioner
ICPC-2: International Classification of Primary Care system
NRS: numeric ranking scale
OR: odds ratio
SEQ: The Standard Evaluation Questionnaire
